# Medical News and Miscellany

**Published:** 1888-09

**Authors:** 


### MEDICAL NEWS AND MISCELLANY.

PERSONAL.—Dr. S. T. Turner, of Marfa, Texas, is at the Polyclinic, N. Y., and directs the JOURNAL to be sent to him at that address for the present.

DR. A. G. CAMP, of Limestone county, the long time representative in the Texas Legislature, was reported, by a telegram received at Austin by Capt. Foster, to be in a dying condition.

MARRIED.—Montgomery—Kelley. In Texarkana, Texas, September 13th (inst.), Dr. A. S. Montgomery, of Tyler, to Miss

Leona S. Kelley, a sister of Mrs. Henry B. Christian, of Texarkana. The JOURNAL'S congratulations are extended to our handsome young friend, and his accomplished bride.

---

P. M. B. A. OF TEXAS.—Assessment No. 8, for one dollar, has been issued from the office of the Physicians' Mutual Benefit Association, for the benefit fund to be provided for the widow and children of the late Dr. Frank A. Tompkins, of Sandy Point, holder of certificate No. 50. We trust members will respond promptly. Last day for payment, October 12th.

---

DEATH OF DR. HOWZE.—The Texas State Medical Association is suffering heavily this year, several of its members having died since the last meeting. The last death of a member reported to us, is that of our talented young friend, Dr. J. T. Howze, of Huntsville. He only joined recently, at the Galveston meeting, in April. Dr. Howze was a graduate of Louisville Medical College, of the class ot 1887. He died at Huntsville, August 30th (ult).

---

PHYSICIANS' MUTUAL BENEFIT ASSOCIATION OF TEXAS.—ASSESSMENT NO. 7—Many of the members have failed, to date, to remit assessment No. 7. Notice is hereby given that the time for remitting—the utmost time allowed by the Constitution and By-laws, sixty days—will expire on the 21st instant, at which time the list will be closed, and all who have not paid this assessment will forfeit their membership, and all monies previously paid in. As a few days remain, we hope delinquents will avail themselves of the opportunity to save themselves.

---

THE TRANSACTIONS T. S. M. ASSOCIATION OF 1888.—This volume has been mailed to members and exchanges. *Delinquents will please forward at least one year's dues* to Treasurer J. Larendon, M. D., Houston, even if they have received the Transactions. For want of means the volume this year, though creditable, under the circumstances, does not begin to compare with that of 1886 and 1887 in mechanical execution nor size; there has been a great falling off in this, corresponding with that in receipts. At Galveston the receipts were $1000 less than at Austin. Shame, gentlemen, shame. Come, pay your dues like men, and don't let the published Transactions bear testimony to your delinquency; your remittance is *needed*, as there was not quite money enough in the treasury to meet the bill, small as it is.

---

MEDICAL DEPARTMENT, UNIVERSITY OF TEXAS.—A meeting of the Regents of the University of Texas was held in Galveston September 3rd, to take steps for the speedy erection of the College building, for which purpose the last Legislature appropriated $50,000. We learn that a plan of the building was agreed upon, to conform to the appropriation,—the sum being inadequate to build such structures as really should be built to be on a scale commensurate with other departments, and with the prospective needs of ten years hence; but,—the site, offered by the city, was thought not eligible, and was rejected. It is situated on the bay, and has no frontage. It is thought to be, altogether, unsuitable for the Medical Department of the University of Texas. The State should purchase a block of ground, in the center of the city and on an elevation, and the Legislature should amend the appropriation so as to make the Medical College an ornament to Galveston, and a credit to Texas.

THE RIGHT MAN IN THE RIGHT PLACE—It gives us great pleasure to learn that our old college-mate and boyhood friend, Dr. Tom. J. McFarland, the quarantine officer at Pass Cavallo, Port Lavaca, has made, since his appointment by State Health Officer Rutherford, a most efficient officer. Constantly at his post, intelligent and ever vigilant, his life free from any and all offenses against morals,—he has established perfect discipline amongst employes, sailors and boatsmen, and has carried out to the letter the rules and regulations of the service. The people of Texas may sleep in peaceful security, so far as any danger is concerned of yellow fever giving McFarland the slip. Dr. Rutherford is to be congratulated on the possession of such *aides* as McFarland, Burke and Wolff,—they are the right men in the right place.

